# Interrelationships between negative mood and clinical constructs: a motivational systems approach

**DOI:** 10.3389/fpsyg.2014.00393

**Published:** 2014-04-30

**Authors:** Gary I. Britton, Graham C. L. Davey

**Affiliations:** ^1^School of Psychotherapy and Psychology, Regent’s University LondonLondon, UK; ^2^School of Psychology, University of SussexBrighton, UK

**Keywords:** anxiety disorders, depression, inflated responsibility, intolerance of uncertainty, negative mood

## Abstract

A series of three experiments was designed to test predictions from a motivational systems approach to understanding the role of clinical constructs in anxiety-based problems. Negative mood, inflated responsibility, and intolerance of uncertainty (IU) were separately manipulated within analog samples to examine their effect on the other two factors. In the first experiment (*n* = 59) the negative mood group scored significantly higher in terms of inflated responsibility than the positive mood group. In the second experiment (*n* = 63) the high responsibility group scored significantly higher in terms of both negative mood and IU than the low responsibility group. In the third experiment (*n* = 61) the high IU group scored significantly higher in terms of negative mood than the low IU group. Tests of indirect effects revealed an indirect effect of IU on inflated responsibility through negative mood and an indirect effect of negative mood on IU through inflated responsibility, suggesting all three constructs are causally interrelated. The findings are consistent with contemporary transdiagnostic views of clinical constructs, and support a view of anxiety that is underpinned by a coordinated and interdependent motivational system evolved to manage threat.

## INTRODUCTION

In contrast to fear, which is an emotion evolved to deal with immediate threats, anxiety is an emotion that has evolved to deal with anticipated threats and challenges (Barlow, 2002). While anxiety has been researched extensively in the clinical psychology and psychopathology literature it has received very little attention in the emotion literature (Tracy and Randles, 2011). This lack of attention has meant that the study of anxiety as a normal functionally adaptive emotion has been relatively neglected when compared with research on clinical anxiety. A theoretical consequence of this is that models of clinical anxiety have had no significant models of normal anxiety to draw on and have therefore tended to reflect models constructed solely around understanding and explaining clinical anxiety itself (Davey, 2006).

How might contemporary cognitive models of clinical anxiety be reconciled with anxiety as an emotion evolved to deal with anticipated threats? Some authors argue anxiety is best viewed as part of an evolved integrated threat management system (e.g., Schaller et al., 2007; Neuberg et al., 2011). Experienced emotions such as anxiety are features of any “precautionary system” that simultaneously alerts the individual to challenges and threats to goals, and coordinates cognitive and behavioral reactions in order that the individual can respond more effectively to these challenges and threats. Threat management systems will evolve separately to deal with specific and different challenges to reproductive fitness, but individual systems will be characterized by a functional coherence in which perceptual, affective, cognitive, and behavioral processes work together to reduce the fitness costs of potential threats (e.g., Frijda, 1986; Keltner et al., 2006). As perceptual, affective, cognitive, and behavioral elements are all part of an integrated evolved functional system, we would expect these elements to be highly coordinated and interdependent, with the affective experience being an emerging property of the activation of the various functional elements in the system (Kenrick and Shiota, 2008; Neuberg et al., 2011).

Can contemporary approaches to understanding clinical anxiety be reconciled with overarching motivational systems approaches (e.g., Griskevicius et al., 2010) and what value might motivational systems approaches add to the understanding of clinical anxiety? If clinical anxiety is fundamentally derived from anxiety as an adaptive emotion then one implication of the motivational systems view of clinical anxiety is that emotional, cognitive and behavioral elements characteristic of anxiety disorders should be coordinated and interdependent within the threat management system relevant to anxiety. The existence of an integrated system underlying anxiety experience should be revealed by experimental studies identifying a network of causal interactions between anxiety-related emotional experience and anxiety-relevant cognitive and behavioral processes. Interactions between the emotional, cognitive, and behavioral elements that make up a system for managing anticipated threats are especially likely to be observed in individuals who chronically perceive themselves to be vulnerable to future threats (e.g., high anxious individuals) or who are made anxious during experimental manipulations (cf. Neuberg et al., 2011).

At this point, however, it is important to understand how clinical psychology researchers have developed their own theoretical approaches to understanding clinically experienced anxiety and how these might be integrated into a functional systems view of anxiety. While the external symptoms of clinical anxiety are well documented and defined (e.g., American Psychiatric Association, 2000) the cognitive features associated with such anxiety have often been explored and described with the use of clinical constructs that attempt to capture the beliefs, attitudes, and thought patterns associated with clinical anxiety and with specific anxiety disorders (Davey, 2003). Within a motivational systems approach it would be expected that the cognitive factors captured by these clinical constructs should represent processes developed to help the individual detect and deal with anticipated threat. Given individuals with anxiety disorders are hypervigilant for threat and especially prepared to react to and deal with anticipated threat, the cognitive constructs developed to explain clinical anxiety are likely to reflect exaggerated examples of the elements that make up any threat management system^1^.

The present paper describes the results of three experiments designed to investigate whether anxiety-related clinical constructs and their affective experience may be representative of a more integrated system underlying anxious responding. We achieved this by taking two example anxiety-relevant constructs, in this case inflated responsibility and intolerance of uncertainty (IU), and one psychopathology relevant mood state, negative mood, and examined the effect of manipulating each one of these factors on measures of the other two. *Inflated responsibility* is defined as the belief that one has the power to bring about or prevent subjectively crucial negative outcomes (Salkovskis, 1985; Rachman, 1998), and *IU* is defined as a “dispositional characteristic that arises from a set of negative beliefs about uncertainty and its connotations and consequences” (Birrell et al., 2011, p. 1200) and is underpinned by appraisals such as “uncertainty is dangerous,” “uncertainty is intolerable,” and “I can’t deal with uncertainty” (Koerner and Dugas, 2006). Inflated responsibility and IU have been extensively researched over the years, and have been highly influential in the development of theories of psychopathology – especially anxiety-based psychopathology.

Considering anxiety as an integrated threat management system within a motivational systems approach is particularly timely because of recent developments in transdiagnostic research conducted on anxiety-relevant clinical constructs. For example, Carleton (2012) has provided compelling evidence that IU is a transdiagnostic construct that is significantly related to measures of a number of anxiety disorders, including generalized anxiety disorder (GAD), obsessive-compulsive disorder (OCD), panic disorder, and social anxiety (Dugas et al., 2005b; Carleton et al., 2007b, 2010; Lind and Boschen, 2009). Furthermore, there is evidence of IU extending beyond anxiety to mediating other mood-related disorders such as depression (McEvoy and Mahoney, 2012). Inflated responsibility is also a construct with transdiagnostic features, having been implicated in the development and maintenance of OCD, GAD, and depressed mood (Aardema et al., 1997; Rachman, 1998; Startup and Davey, 2003). While these studies indicate that clinically developed constructs have a much broader application across anxiety and mood disorders than was previously suspected, there is little or no research to date on how these constructs interrelate. If these constructs interrelate through an evolutionarily determined threat management system, then we would expect them to be highly coordinated and interdependent, and to influence the experience of negative mood (e.g., anxiety or depression) in experimental studies designed to independently manipulate each variable. Specifically, if constructs, emotion states and symptoms are all components of a functionally integrated threat management system evolved to deal with anticipated threats, then we would predict that (1) manipulation of individual constructs would either directly or indirectly increase self-reported experience of the relevant emotion (i.e., negative mood^2^), that (2) manipulation of negative mood would increase scores either directly or indirectly on construct measures, and (3) manipulation of each construct would increase scores either directly or indirectly on the other.

## EXPERIMENT 1

The first experiment is designed to assess the effect of an experimental mood manipulation on self-report measures of inflated responsibility and IU. Participants were randomly assigned to either a positive or negative mood manipulation group and underwent appropriate music-based mood manipulation procedures before being asked to complete a short questionnaire consisting of items relating to the constructs inflated responsibility and IU. Because specific discrete mood manipulations are difficult to achieve in practice (Gross and Levenson, 1995; Rottenberg et al., 2007; Meeten and Davey, 2012) we adopted a polar, non-specific valenced mood manipulation using mood manipulations that have previously been used in studies investigating the role of mood in anxious psychopathology (e.g., MacDonald and Davey, 2005). Negative mood encompassing both feelings of anxiety and sadness (depression) is a common feature of OCD (Roper and Rachman, 1976; Salkovskis, 1985; Frost et al., 1986) and GAD (Metzger et al., 1990; Meyer et al., 1990; Davey et al., 1992). If cognitive constructs such as inflated responsibility and IU represent cognitive components of an integrated threat management system then we would predict that negative mood should be accompanied, either directly or indirectly, by facilitated scores on measures of inflated responsibility and IU. If the constructs of inflated responsibility and IU represent unidirectional causes of negative mood and anxiety-related symptoms, however, we would not predict an effect of negative mood on measures of these constructs.

### METHOD

#### Participants

Participants were 59 psychology undergraduates from the University of Sussex (men: 7; women: 52). Age ranged from 18 to 43 years (*M* = 21.03, SD = 5.61). All of the participants were volunteers who received partial fulfillment of a course requirement by taking part in the experiment. There were no significant differences between the two groups in terms of age, *t*(57) = 0.97, *p* = 0.33, or the distribution of male participants, χ^2^(1) = 0.13, *p* = 0.72, suggesting randomization between groups was successful.

Ethical approval for experiment 1 was provided by the ethics committee at The University of Sussex.

#### Procedure

Participants were randomly assigned to one of two groups, depending on the valence of the mood manipulation they were to receive, these groups were labeled positive (*n* = 30) and negative (*n* = 29). Randomization was achieved by the experimenter drawing lots prior to the participant’s arrival. All participants were tested individually in a small room containing a PC with headphones and an angle-poise lamp. There was a retractable blind over the only window in the room which could be open or closed (closing of the blind almost completely stopped day light from entering the room). Participants completed a consent form which stated the experiment was about music comprehension and memory and how this is related to personality. The consent form informed participants that they would be asked to listen to some music and then, after a ten minute break, they would be asked to fill in some questionnaires.

***Stage 1: mood manipulation***. Participants wore headphones so they could listen to a short piece of music. The music lasted approximately 8 min. The experimenter left the room while the music was playing and returned after 8 min. Participants in the negative mood group listened to a piece of music which had previously been shown to induce a negative mood state (MacDonald and Davey, 2005): Gyorgy Ligeti, *Lux Aeterna*. Blinds were drawn over the windows and the main room lights were switched off, only the angle-poise lamp was used to illuminate the room. Participants in the positive mood group listened to a different piece of music: Delibes, *Mazurka* from *Coppelia* (only the section from 1 min 46 s to 3 min 10 s, looped). The blinds in the room were left open allowing full day light into the room, the main lights were turned on as was the angle-poise lamp.

***Stage 2: 10 min break and short questionnaire***. The experimenter re-entered the room immediately after the music had finished and reminded the participant about the impending 10 min break before asking the participant if they would mind filling in a questionnaire unrelated to the experiment during the break. The experimenter told the participant the questionnaire was related to a separate questionnaire study being conducted by the experimenter’s supervisor and that the questionnaire would take just over 5 min to complete. All participants agreed to fill in the questionnaire. The experimenter left the room for 10 min while the participant filled in the questionnaire. The data collected in this questionnaire was actually to be used in the analysis of the present study. The reason for deceiving participants about this questionnaire was to reduce any experimental demand effects and to minimize any perceived link between the music as a mood manipulation procedure and subsequent data collection. The short questionnaire contained a separate consent form which stated the questionnaire study was broadly concerned with decision making. Mood was measured in the questionnaire using three questions where participants were asked to rate their current level of sadness, happiness and anxiety on separate 100 point visual analog scales (VAS) (where 0 = *not at all sad/happy/anxious* and 100 *extremely/sad/happy/anxious*). Responsibility was measured using three example items taken from the Responsibility Attitude Scale (RAS; Salkovskis et al., 2000). The items were “If I think bad things, this is as bad as DOING bad things,” “I will be condemned for my actions,” and “Other people should not rely on my judgment.” Participants rate how much they agree with each statement at the present moment in time on separate 100 point VAS. The three items had acceptable internal consistency (α = 0.78). These items were chosen as they seemed to capture in a small number of items many of the key characteristics of inflated responsibility (see, e.g., Salkovskis et al., 1996). IU was measured using three items taken from the IU scale (IUS; Freeston et al., 1994). Participants rate to what extent they agree with the items at this exact moment in time on three separate 100 point VAS (where 0 = *Totally Disagree* and 100 = *Totally Agree*). The items were “Uncertainty stops me having a firm opinion,” “It’s unfair there are no guarantees in life,” and “Being uncertain means I am not first rate.” The three items had acceptable internal consistency (α = 0.80). These items were chosen as they seemed to capture in a small number of items many of the key characteristics of IU (see, e.g., Freeston et al., 1994).

***Stage 3: full questionnaires and debrief***. The experimenter re-entered the room after the 10 min “break” had finished and gave the participant another questionnaire booklet. The participant was asked to inform the experimenter when they had finished the questionnaire booklet. The questionnaire booklet contained a number of questionnaires. The first questionnaire was a “music comprehension and memory” questionnaire designed specifically for the purposes of this experiment and was not used in the data analysis of this experiment. As part of the questionnaire booklet participants completed the full version of the RAS (Salkovskis et al., 2000) and the full version of the IUS (Freeston et al., 1994). After completing the questionnaire booklet, participants were thanked and debriefed, and any participant who had undergone a negative mood manipulation was offered a positive mood manipulation before they left.

### STATISTICAL ANALYSES

Within this paper, two groups were consider to differ on a given construct only if the VAS measure of that construct (if more than one question was used, then a composite of these VAS measures) reached the level of *p* < 0.05 (two-tailed) and if the VAS measure of the construct correlated significantly with the full trait measure of the construct (note that only full trait measures of IU and inflated responsibility were measured in the three experiments reported in this paper, no “trait” measures of mood were used and so only VAS measures were considered in relation to mood). In all three experiments reported in this paper the reliability of the full measure of inflated responsibility: the RAS, and IU: the IUS, were excellent (lowest alphas for both measures α = 0.91, respectively, in Experiment 3). In addition, in all three experiments reported in this paper the VAS measures of inflated responsibility and IU were highly significantly positively correlated with their respective full trait measure (RAS or IUS) in each experiment (all correlations significant at *p* < 0.001). Finally, across all three experiments, where a significant difference was found between the two respective groups on the VAS measures of inflated responsibility or IU, a significant difference between the respective groups was also found on the full trait measure of that construct (RAS or IUS). Where a significant difference was not found between the two respective groups on the VAS measures of inflated responsibility or IU, significant differences between the respective groups were also not found on the full trait measures of that construct (RAS or IUS). Given the consistency of these results, and in an effort to be succinct, reliability data for the RAS and IUS, correlations between VAS measures and the RAS and IUS and group differences on the RAS and IUS are not reported in any of the experiments that follow. It is worth noting, however, that the fact all significant differences between groups on VAS measures of inflated responsibility and IU were mirrored with significant differences on the full trait measures of those constructs (RAS and IUS) provides a good deal of confidence in the validity of these findings, given that these full measures of constructs are trait measures which are thought to be difficult to influence by experimental manipulation.

It should be noted that a short-form version of the IUS exists which it has been argued better approximates IU as a construct independent from worry than the original IUS (Carleton et al., 2007a). The short-form IUS consists of 12 items which are identical to 12 items found in the original IUS. Given the argument that the short-form IUS better approximates IU as a construct independent from worry, all analyses across all three experiments which involved the original IUS were rerun. In these analyses only the 12 items found in the IUS short-form were included. The results of these analyses were identical in terms of significant/non-significant distinctions to those reported above for the original IUS.

### RESULTS

#### Mood manipulation check

Each mood measure was subjected to an independent measures sample *t*-test. For the three mood measures, the assumption of homogeneity of variances was violated, and so adjusted *p*-values are reported. The negative mood group (*M* = 30.65, SD = 22.23) scored significantly higher on the sadness measure than the positive mood group (*M* = 09.57, SD = 08.07), *t*(35.03) = 4.81, *p* < 0.001, *d* = 1.080. The positive mood group (*M* = 73.90, SD = 10.33) scored significantly higher on the happiness measure than the negative mood group (*M* = 56.79, SD = 19.46), *t*(42.36) = 4.20, *p* < 0.001, *d* = 1.144. The negative mood group (*M* = 37.53, SD = 26.48) scored significantly higher on the anxiety measure than the positive mood group (*M* = 18.20, SD = 17.28) *t*(47.96) = 3.32, *p* < 0.01, *d* = 0.834. These data suggest that participants in the negative mood group were significantly more anxious and sad, and less happy than participants in the positive mood group.

#### Responsibility measures

A composite responsibility score was created by combining the means of the three questions used in the short questionnaire. The negative mood group (*M* = 35.83, SD = 19.47) scored significantly higher on this composite responsibility measure than the positive mood group (*M* = 25.72, SD = 17.97), *t*(57) = 2.07, *p* < 0.05, *d* = 0.612.

#### IU measures

A composite IU score was created by combining the means of the three questions measuring IU used in the short questionnaire. The negative mood group (*M* = 41.35, SD = 19.21) scored higher on this composite IU measure than the positive mood group (*M* = 32.28, SD = 19.23) but this difference was not significant, *t*(57) = 1.81, *p* = 0.08, *d* = 0.479.

### SUMMARY

The findings from Experiment 1 indicate that participants undergoing a negative mood manipulation scored significantly higher in terms of inflated responsibility than participants undergoing a positive mood manipulation. The mood manipulation had no statistically significant direct effect on IU (however, see the section on meditational analyses and tests of indirect effects for evidence of a significant indirect effect of negative mood on IU).

## EXPERIMENT 2

The second experiment is designed to assess the effect of an experimental inflated responsibility manipulation on self-report measures of negative mood and IU. This novel procedure uses a vignette-based responsibility manipulation prior to estimating the manipulations effect on mood and IU.

### METHOD

#### Participants

Participants were 63 psychology undergraduates from the University of Sussex (men: 6; women: 57). Age ranged from 18 to 56 years (*M* = 22.06, SD = 7.45). All of the participants were volunteers who received partial fulfillment of a course requirement by taking part in the experiment. There were no significant differences between the two groups in terms of age, *t*(61) = 0.71, *p* = 0.48, or the distribution of male participants, χ^2^(1) = 0.67, *p* = 0.41, suggesting randomization between groups was successful.

Ethical approval for Experiment 2 was provided by the ethics committee at The University of Sussex.

#### Procedure

Participants were randomly assigned to one of two groups, depending on the manipulation they were to receive. These groups were labeled high responsibility (*n* = 31) and low responsibility (*n* = 32). Randomization was achieved by the experimenter drawing lots prior to the participant’s arrival. All participants were tested individually in a small room that simply contained a chair and a desk. Participants completed an informed consent form which stated participants would be asked to read a “true story” and that they would be asked some questions about the story. The consent form informed participants there would then be a ten-minute break and that they would then be asked to fill in some questionnaires.

***Stage 1: responsibility manipulation***. Participants read one of two stories, dependent on the group to which they had been assigned. Both pieces were written from an autobiographical perspective by a character called Clara, a 25 year old woman. Although participants were told they were reading a true story the stories were in fact fictitious and were created for the experiment. Both stories were printed on A4 paper and were of a similar length (about three and a half sides of A4). In the story given to the high responsibility group Clara is a person who lacks any sense of responsibility. Clara describes her lack of responsibility and gives descriptions of incidences that she was partly or wholly responsible for but over which she had failed to take any responsibility. Clara describes how her failure to take responsibility for these incidences (and others) had led to negative consequences for her. Throughout the story Clara expresses regret about her lack of responsibility and toward the end of the story states that she wants to become a more responsible person but that she feels she needs some help in doing this. Participants in the high responsibility group are asked to write down advice to help Clara feel, and act, like a more responsible person. “Example advice,” consisting of five statements, is given after the story to give participants some idea of the sort of advice they may want to offer. In the story given to the low responsibility group Clara is a person who has an inflated sense of responsibility. Clara describes her inflated sense of responsibility and gives descriptions of incidences which have occurred which she had little or no control over but over which she felt immense responsibility. Clara describes how her inflated sense of responsibility has had negative consequences for her. Throughout the story Clara displays an awareness regarding the negative affect her inflated sense of responsibility is having on her life and toward the end of the story states that she wants to become a person who feels less responsible but that she feels she needs some help in doing this. Participants in the low responsibility group are asked to write down advice to help Clara feel less responsible. “Example advice,” consisting of five statements, is given after the story to give participants some idea of the sort of advice they may want to offer.

To ensure the responsibility manipulation complied with definitions of the inflated responsibility construct (e.g., Salkovskis et al., 1996) four specific features of inflated responsibility were defined and explicitly referred to in the advice vignettes given to participants. These features were: (1) a sense of feeling overly responsible in the most literal sense (i.e., feeling bad for harm caused, taking on responsibility for things that are not necessarily the individual’s fault), (2) the idea that thinking about something (e.g., causing harm) is as bad as doing something, (3) the idea that not preventing harm is as bad as causing harm, (4) worrying about causing harm before anything has actually happened (e.g., hypervigilance). In the story given to the low responsibility group Clara displays all of these defined features while the “example advice” offered to Clara is aimed at advising her about how to minimize or eliminate these feelings from her life. In the story given to the high responsibility group Clara is described as not displaying any of these defined features and displays a range of opposing feelings. The “example advice” offered to Clara is aimed at advising her about how she can, and should, bring these feelings into her life. After reading their respective vignettes, participants in both groups were asked to write their advice on A4 paper. The experimenter left the room while the participant read the story and wrote down their advice. No set time limit was given to complete this task. The responsibility manipulation is based on Bem’s self-perception theory that proposes that an individual will infer his or her attitude based on information derived from his or her behavior (Bem, 1972). Salancik and Conway (1975) proposed that the individual will infer his or her attitude through a process of generating and assessing relevant information from the past and present, and that the individual will be especially likely to use information made most conspicuous to them at the time. When an individual describes an attitude or behavior positively or negatively, therefore, he or she will generate cognitions consistent with their endorsement.

***Stage 2: 10 min break and short questionnaire***. As in stage 2 of Experiment 1, participants were asked to fill in a short questionnaire participants were told was related to a separate questionnaire study. The questionnaire contained the VAS measures of the three constructs. Mood, sadness, and anxiety were measured in the questionnaire using the same questions used in Experiment 1 but an additional question was added, participants were now also asked to rate their current level of negativity on a 100 point VAS (where 0 = *not at all negative* and 100 = *extremely negative*). The three items measuring negative mood had good internal consistency (α = 0.86). IU was measured using five items taken from the IUS (Freeston et al., 1994). Participants rate to what extent they agreed with the items at that exact moment in time on five separate 100 point VAS (where 0 = *Totally Disagree* and 100 = *Totally Agree*). The five items were, “Uncertainty stops me from having a strong opinion,” “Uncertainty makes life intolerable,” “I can’t stand being taken by surprise,” “I can’t stand being undecided about my future,” and “Being uncertain means that I am not first rate,” The five items had moderate internal consistency (α = 0.69). These items were chosen as they seemed to capture in a small number of items many of the key characteristics of IU (see, e.g., Freeston et al., 1994). Responsibility was measured using four items each taken from the RAS (Salkovskis et al., 2000) where participants rate how much they agree with each statement on separate 100 point VAS. These items were “I often take responsibility for things that other people do not think are my fault,” “Even if my actions are unlikely to bring about negative consequences for others, I should always try to prevent them from occurring,” “If I think bad things, this is as bad as DOING bad things,” and “To me, not acting to prevent disaster is as bad as making disasters happen.” These four items were used because they each separately related to the four defined inflated responsibility characteristics portrayed in the experimental vignettes and seemed to capture in a small number of items many of the key characteristics of inflated responsibility (see, e.g., Salkovskis et al., 1996). The four items appeared to have moderate internal consistency (α = 0.66).

***Stage 3: full questionnaires and debrief***. The experimenter re-entered the room after the 10 min “break” had finished and then gave the participant another questionnaire booklet and asked the participant to inform the experimenter when they had finished the questionnaire booklet. The experimenter then left the room until the participant had finished the questionnaire booklet. This questionnaire booklet contained the full version of the RAS (Salkovskis et al., 2000) and the full IUS (Freeston et al., 1994). After completing the questionnaire booklet, participants were thanked and debriefed.

### RESULTS

#### Responsibility manipulation check

A composite responsibility score was created by combining the means of the four questions measuring responsibility used in the short questionnaire. The high responsibility group (*M* = 54.11, SD = 13.55) scored significantly higher on the composite measure of responsibility than the low responsibility group (*M* = 33.95, SD = 17.76), *t*(61) = 5.13, *p* < 0.001, *d* = 1.314.

#### Mood measures

The high responsibility group (*M* = 34.45, SD = 21.46) scored significantly higher on the sadness measure than the low responsibility group (*M* = 14.75, SD = 13.01), *t*(49.15) = 4.39, *p* < 0.001, *d* = 1.252 (as the assumption of homogeneity of variances was violated the adjusted *p*-value is reported for this finding). The high responsibility group (*M* = 39.35, SD = 25.52) scored significantly higher on the anxiety measure than the low responsibility group (*M* = 26.94, SD = 21.08), *t*(61) = 2.11, *p* < 0.05, *d* = 0.540. The high responsibility group (*M* = 35.48, SD = 24.73) scored significantly higher on the negativity measure than the low responsibility group (*M* = 15.43, SD = 14.80), *t*(48.75) = 3.89, *p* < 0.001, *d* = 1.114 (as the assumption of homogeneity of variances was violated the adjusted *p*-value is reported for this finding). The high responsibility group (*M* = 36.43, SD = 21.11) scored significantly higher on the composite measure of negative mood than the low responsibility group (*M* = 19.04, SD = 13.52), *t*(50.81) = 3.88, *p* < 0.001, *d* = 1.089 (as the assumption of homogeneity of variances was violated the adjusted *p*-value is reported for this finding).

In summary, these data suggest that the high responsibility group were significantly more anxious, sadder, and more negative (both on VAS and composite measures) than the low responsibility group. In order to assess whether the two differing vignettes may have directly affected mood by containing differential levels of negative material, eight independent participants (men: 2; women: 6; age: *M* = 24.38, SD = 3.89) were asked to read each vignette (only the story part without the subsequent advice examples) and to report on a 100-point VAS scale how the vignette made them feel (where 0 = extremely positive, 50 = neither positive or negative, and 100 = extremely negative). A repeated measures *t*-test indicated that those reading the low responsibility vignette reported significantly more negativity (*M* = 62.75, SD = 8.21) than those reading the high responsibility vignette (*M* = 53.38, SD = 5.63), *t*(7) = 2.61, *p* < 0.05, *d* = 1.331. Given that the low responsibility manipulation appears to contain more negative material than the high responsibility manipulation it appears highly unlikely the high responsibility group experienced significantly greater negative mood than the low responsibility group due to differences in the respective vignettes’ content.

#### IU measures

A composite IU score was created by combining the means of the five questions measuring IU used in the short questionnaire. The high responsibility group (*M* = 41.55, SD = 16.88) scored significantly higher on this composite IU measure than the low responsibility group (*M* = 32.11, SD = 15.11), *t*(61) = 2.34, *p* < 0.05, *d* = 0.599.

### SUMMARY

Experiment 2 confirmed that manipulating inflated responsibility using a vignette-based responsibility manipulation had a consistent effect on self-reported mood. Specifically, the high responsibility group generated significantly higher self-reported ratings of anxiety, sadness, negativity, and general negative mood (as measured by the composite negative mood measure) than the low responsibility group. Subsequent ratings of the vignettes themselves by independent raters revealed that this differential effect on mood could not be accounted for by any inherent differences in negative content between the high and low responsibility vignettes, suggesting that mood differences were caused by the responsibility-related cognitions generated by the advice participants provided after reading the vignettes. The high responsibility group also reported significantly higher levels of IU than the low responsibility group.

## EXPERIMENT 3

The third experiment is designed to assess the effect of an experimental IU manipulation on self-report measures of negative mood and inflated responsibility. This novel procedure, which is similar to the responsibility manipulation used in Experiment 2, uses a vignette-based IU manipulation prior to estimating the manipulations effect on inflated responsibility and negative mood.

### METHOD

#### Participants

Participants were 61 undergraduates from the University of Sussex (men: 16; women: 45). Age ranged from 18 to 33 years (*M* = 20.26, SD = 2.82). All of the participants were volunteers who received partial fulfillment of a course requirement or received a small monetary fee for taking part in the experiment. There were no significant differences between the two groups in terms of age, *t*(59) = 0.91, *p* = 0.36, or the distribution of male participants, χ^2^(1) = 2.80, *p* = 0.11, suggesting randomization between groups was successful.

Ethical approval for Experiment 3 was provided by the ethics committee at The University of Sussex.

#### Procedure

***Stage 1: IU manipulation***. Participants were randomly assigned to one of two groups, the high IU group (*n* = 30) and the low IU group (*n* = 31). Randomization was achieved by the experimenter drawing lots prior to the participant’s arrival. Participants read one of two stories, dependent on the group to which they had been assigned. Both pieces were written from an autobiographical perspective by a character called Kayla, a 25 year old woman. Although participants were told they were reading a true story the stories were in fact fictitious and were created for the experiment. Both stories were printed on A4 paper and were of a similar length (about two sides of A4). In the story given to the high IU group Kayla is a person who has very little or no reaction to uncertainty. In the story Kayla discusses her inability to react to uncertainty and gives descriptions of uncertain situations she has had little or no reaction too. Kayla describes how her failure to respond to the uncertainty surrounding these situations (and others) has led to negative consequences for herself and others. Throughout the story Kayla expresses regret about her inability to react to uncertainty and toward the end of the story states that she wants to become someone who reacts when faced with uncertain situations as this may prevent her from making risky decisions, but states that she needs some help in doing this. Participants in the high IU group are asked to write down advice to help Kayla react better to uncertainty. “Example advice,” consisting of five statements, is given after the story to give participants some idea of the sort of advice they may want to offer. In the story given to the low IU group Kayla is a person who finds uncertainty very difficult to cope with as it causes her a lot of stress and anxiety. In the story Kayla describes how uncertain situations worry her and gives descriptions of uncertain situations which have paralyzed her and prevented her from reacting. Kayla describes how her failure to act in these situations (and others) due to her fear of uncertainty has led to negative consequences for herself and others. Throughout the story Kayla displays an awareness regarding the negative affect her anxiety surrounding uncertainty is having on her life and toward the end of the story states that she wants to become a person who is able to relax when faced with an uncertain situation and that she does not want to be paralyzed by uncertainty, but that she feels she needs some help in doing this. Participants in the low IU group are asked to write down advice to help Kayla become less anxious and paralyzed by uncertainty. “Example advice,” consisting of five statements, is given after the story to give participants some idea of the sort of advice they may want to offer.

To ensure that the IU manipulation complied with definitions of the IU construct, five specific features of IU were defined and explicitly referred to in the advice vignettes given to participants. These features were: (1) uncertainty leads to the inability to act, (2) uncertainty is stressful and upsetting, (3) unexpected events are negative and should be avoided, (4) being uncertain is unfair, (5) being uncertain reflects badly on a person (see Freeston et al., 1994). In the story given to the low IU group Kayla displays all of these beliefs about uncertainty while the “example advice” offered to Kayla is aimed at advising her about how to minimize or eliminate these kinds of beliefs about uncertainty. In the story given to the high IU group Kayla does not display any of these beliefs about uncertainty and in fact displays a range of opposing beliefs. The “example advice” offered to Kayla is aimed at advising her about how she can, and should, bring these kinds of beliefs about uncertainty into her life. After reading their respective vignettes, participants in both groups were asked to write their advice on A4 paper. The experimenter left the room while the participant read the story and wrote down their advice. No set time limit was given to complete this task.

***Stage 2: 10 min break and short questionnaire***. As in stage 2 of Experiments 1 and 2, participants were asked to fill in a short questionnaire participants were told was related to a separate questionnaire study. The questionnaire contained the VAS measures of the three constructs. IU was measured in the short questionnaire using the same five questions as were used in experiment two. The five items had acceptable internal consistency (α = 0.75). Mood was measured in the questionnaire using the same three questions as Experiment 2. The three items measuring negative mood had mediocre internal consistency (α = 0.66). Responsibility was measured in the short questionnaire using the same four questions as were used in experiment two. These four items also had mediocre internal consistency (α = 0.63).

***Stage 3: full questionnaires and debrief***. The experimenter re-entered the room after the 10 min “break” had finished and gave the participant another questionnaire booklet, asking the participant to inform the experimenter when they had finished the questionnaire booklet. The experimenter then left the room until the participant had finished the questionnaire booklet. This questionnaire booklet contained the full version of the RAS (Salkovskis et al., 2000) and the IUS (Freeston et al., 1994). After completing the questionnaire booklet, participants were thanked and debriefed.

### RESULTS

#### IU manipulation check

A composite IU score was created by combining the means of the five questions measuring IU used in the short questionnaire. The high IU group (*M* = 33.93, SD = 14.55) scored significantly higher on this composite IU measure than the low IU group (*M* = 16.89, SD = 11.49), *t*(59) = 5.10, *p* < 0.001, *d* = 1.300.

#### Mood measures

The high IU group (*M* = 23.80, SD = 19.30) scored higher on the sadness measure than the low IU group (*M* = 19.39, SD = 15.99), but this difference was not significant, *t*(59) = 0.97, *p* = 0.35, *d* = 0.249. The high IU group (*M* = 36.63, SD = 24.90) scored significantly higher on the anxiety measure than the low IU group (*M* = 23.13, SD = 22.63), *t*(59) = 2.22, *p* < 0.05, *d* = 0.567. The high IU group (*M* = 34.20, SD = 21.64) scored higher on the negativity measure than the low IU group, (*M* = 24.36, SD = 21.01), but this difference was not significant, *t*(59) = 1.80, *p* = 0.08, *d* = 0.461. A composite negative mood score was created by combining the means of the three questions measuring negative mood used in the short questionnaire. The high IU group (*M* = 31.54, SD = 15.82) scored significantly higher on this composite measure of negative mood than the low IU group (*M* = 22.29, SD = 16.27), *t*(59) = 2.25, *p* < 0.05, *d* = 0.576.

In summary, the high IU group scored significantly higher in terms of anxiety and general negative mood (as measured by the negative mood composite measure) than the low IU group. In order to assess whether the two differing vignettes may have directly affected mood by containing differential levels of negative material, nine independent participants (men: 1; women: 8; age: *M* = 23.46, SD = 3.49) were asked to read each vignette (only the story part without the subsequent advice examples) and to report on a 100-point VAS scale how the vignette made them feel (where 0 = extremely positive, 50 = neither positive or negative, and 100 = extremely negative). A repeated measures *t*-test indicated that those reading the low IU vignette reported more negativity (*M* = 61.89, SD = 13.74) than those reading the high IU vignette (*M* = 54.44, SD = 17.61) but this difference was not significant, *t*(8) = 1.01, *p* = 0.34, *d* = 0.471.

#### Responsibility measures

A composite responsibility score was created by combining the means of the four questions measuring responsibility used in the short questionnaire. The high IU (*M* = 38.05, SD = 16.57) group scored higher on this composite responsibility measure than the low IU group (*M* = 32.35, SD = 17.77), but this difference was not significant, *t*(59) = 1.30, *p* = 0.20, *d* = 0.332.

### SUMMARY

In summary, the high IU group scored significantly higher than the low IU group in terms of anxiety and composite negative mood but not inflated responsibility (however, see the section on meditational analyses and tests of indirect effects for evidence of a significant indirect effect of IU on inflated responsibility).

### MEDIATION ANALYSIS AND TESTS OF INDIRECT EFFECTS

**FIGURE 1:**
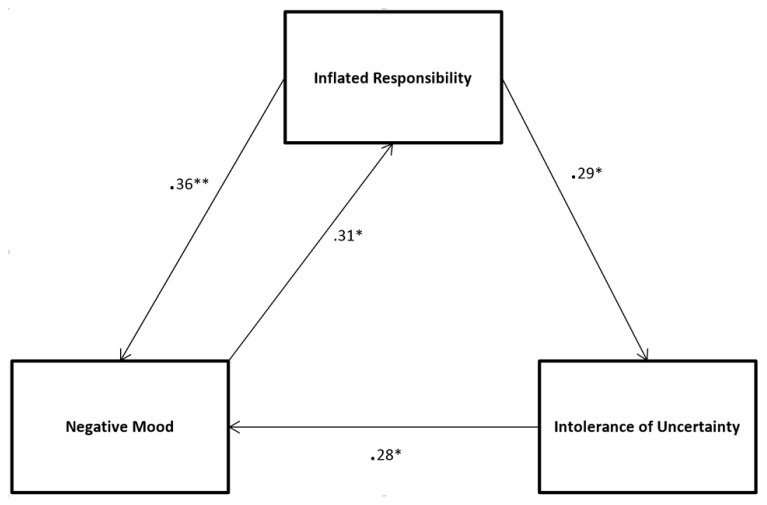
**Significant direct relationships between the three constructs to emerge from Experiments 1–3.** Standardized beta values and their significance (*p < 0.05, **p < 0.01) are reported. Note that significant indirect effects are not shown.

The model of the direct significant relationships between negative mood, inflated responsibility and IU to emerge from Experiments 1–3 is graphically presented in **Figure 1**. If the manipulation of a given construct in an experiment led to a significant direct effect on another construct then this is indicated in **Figure 1** by a causal line joining the two constructs together. Mediation analysis and tests of indirect effects were performed for two purposes: (A) to examine if any direct relationship between variable *x* and variable *y* was partially or fully mediated by variable *m* (as suggested by the model in **Figure 1**) and (B) to see if variable *x* might have an indirect effect on variable *y* through variable *m* (as suggested by the model in **Figure 1**).

In all the analyses which follow the *x* variable used in each analysis is the grouping variable used to separate the high and low group on the given *x* construct in the relevant experiment in which construct *x* was manipulated. The *m* and *y* variables used in each analysis were the composite VAS score for the respective *m* and *y* constructs in the relevant experiment in which construct *x* was manipulated. Analyses were performed using the statistical package AMOs 21. Bootstrapping (1000 samples) was used to evaluate the significance of the indirect pathways.

### MEDIATION ANALYSIS

Examination of **Figure 1** suggests that the causal relationship between inflated responsibility (*x*) and negative mood (*y*) may be partially or fully mediated by IU (*m*). Before the effect of the mediator was taken into account, inflated responsibility significantly predicted negative mood (β = 0.45, *p* < 0.001). Although the relationship between inflated responsibility and negative mood was partially mediated by IU this relationship remained significant when the mediator was taken into account (β = 0.36, *p* < 0.01). The path therefore is kept in the model.

### TESTS OF INDIRECT EFFECTS

Examination of **Figure 1** also suggests three potential indirect casual pathways through which one construct may be influencing another. Firstly, inflated responsibility (*x)* may have an indirect causal effect on negative mood (*y)* through IU (*m*). Secondly, IU (*x*) may have an indirect causal effect on inflated responsibility (*y*) through negative mood (*m*). Finally, negative mood (*x*) may have an indirect causal effect on IU (*y*) through inflated responsibility (*m*).

The indirect effect of inflated responsibility on negative mood through IU was significant (*z* = 0.08, *p* < 0.05) suggesting inflated responsibility has both a significant direct and indirect effect on negative mood. The indirect pathway connecting IU to inflated responsibility through negative mood was also significant (*z* = 0.11, *p* < 0.05) suggesting that while IU may not have a significant direct casual effect on inflated responsibility (see Experiment 3), IU has a significant indirect causal influence on inflated responsibility through its causal effect on negative mood. Finally, the indirect pathway connecting negative mood to IU through inflated responsibility was significant (*z* = 0.17, *p* < 0.05) suggesting that while negative mood may not have a significant direct casual effect on IU (see Experiment 1), negative mood has a significant indirect causal influence on IU through its causal effect on inflated responsibility. In summary, the direct causal relationship between inflated responsibility and negative mood remained significant even after the mediating effect of IU was taken into account. In addition to the significant direct causal pathways connecting negative mood, inflated responsibility and IU depicted in **Figure 1**, three significant indirect causal pathways also connect the constructs: firstly, inflated responsibility has a significant indirect effect on negative mood through IU, secondly, IU has a significant indirect effect on inflated responsibility through negative mood and, finally, negative mood has a significant indirect effect on IU through inflated responsibility.

## DISCUSSION

The purpose of this series of experiments was to demonstrate the integrated systems-like nature of the constructs and affective experience postulated to underlie some common forms of clinical anxiety. In three experiments we manipulated two clinical constructs (inflated responsibility and IU) and one affective state (negative mood) to determine the direct effect of these manipulations on each of the other measures. Finally, the causal pathways between these variables were confirmed using mediational analyses and tests of indirect effects were conducted, and the causal model to emerge from these processes is shown in **Figure 1**. Not shown in **Figure 1** is the indirect effect of inflated responsibility on negative mood through IU; the indirect effect of IU on inflated responsibility through negative mood and, finally, the indirect effect of negative mood on IU through inflated responsibility.

The model presented in **Figure 1**, when indirect effects are taken into consideration, supports a view of the constructs used to explain clinical anxiety as representing elements of an integrated system underlying anxious responding. Higher scores on both clinical constructs give rise to increased negative mood, as would be predicted or at least implied in most accounts adopting these factors as explanatory constructs (e.g., Dugas et al., 1998; Salkovskis and Freeston, 2001). Higher scores on negative mood also facilitates – either directly or indirectly – measures of both clinical constructs, and as a consequence will prime the cognitive processes inherent in these constructs (e.g., assessing one’s ability to bring about or prevent negative outcomes, appraising uncertainty as dangerous or intolerable). The findings presented in this paper are consistent with clinically relevant emotions such as anxiety being considered as part of an evolved integrated threat management system that alerts the individual to threats to goals or challenges, and coordinates cognitive, behavioral, and affective reactions to enable the individual to respond more effectively to these threats and challenges. Rather than one set of factors (e.g., constructs) being causes of a different set of factors (e.g., affect), they are all integrated components of an anxiety precautionary system that promotes a “cascade” of relevant perceptions, cognitions, behaviors, and affective experience conducive to solving the adaptive problem (Kenrick et al., 2010). The findings are also consistent with recent experiments demonstrating how anxiety-related clinical symptoms (e.g., OCD relevant aversive intrusive thoughts) facilitate anxiety-relevant appraisal processes such as IU and responsibility – indicating a bidirectional relationship between actual anxiety symptoms and OCD-relevant clinical constructs and appraisal processes (Davey et al., 2013).

The integrated, systems-like nature of the model derived from these findings is also consistent with contemporary transdiagnostic views of clinical constructs, which argue that the phenomena measured by clinical constructs have evolutionary origins based on the identification and amelioration of threats (e.g., Carleton, 2012). For example, uncertainty is more than just a trigger for anxiety, it is itself considered threatening (Epstein, 1972), can facilitate the perception of threat (Dugas et al., 2005a) and activate biologically relevant responses to threat such as the startle response (Nelson and Shankman, 2011). These findings suggest that clinically relevant constructs have effects beyond clinical symptoms, and also influence cognitive, perceptual, and biological responses in a way that would be predicted by an evolutionarily conceptualized motivational systems approach. In the case of IU, the ability to tolerate uncertainty is a common dispositional characteristic with dimensional characteristics across both non-clinical and clinical populations (Norton, 2005; Carleton et al., 2012b), suggesting that it is an evolved characteristic that differs in intensity across individuals and becomes most predominant in clinical populations exhibiting disorders of either anxiety or mood. Our findings also have implications for models of anxiety that use constructs such as inflated responsibility and IU as a central explanatory construct. For example, while the finding that inflated responsibility directly effects negative mood is consistent with the predictions of cognitive models of OCD that have inflated responsibility as a central causal construct (e.g., Rachman, 1997; Salkovskis and Freeston, 2001) the finding that negative mood commonly experienced by OCD sufferers may be a direct causal factor in elevating inflated responsibility beliefs suggests that negative mood is not simply a consequence of cognitions associated with inflated responsibility as has been implied by these models. Models which adopt inflated responsibility as a central explanatory construct need to account for the bidirectional nature of this construct’s relationship with negative mood.

The integrated nature of the relationships between inflated responsibility, IU and negative mood might be expected if these clinical constructs encompass adaptive elements that help the individual to identify and manage threats, and these processes are activated by anxiety-generating events. Having been developed in many cases from clinical experience to help understand clinical disorders, however, clinical constructs may often confuse in their definitions a range of cognitive processes that span both adaptive threat management processes and less adaptive responses to threat that generate anxiety and negative affect and are directly symptom relevant. For example, inflated responsibility encompasses the adaptive belief that one has the power to prevent harm, but even low risk threats are then seen as essential to prevent and are anxiety generating (Salkovskis, 1985). IU embraces the adaptive desire for predictability but the less adaptive “paralysis of cognition and action” in the face of uncertainty (Birrell et al., 2011). While clinical constructs may encompass cognitive and behavioral processes that contribute adaptively to dealing with threat, by their very nature they may also encompass those processes that give rise to exaggerated affective reactions causing distress. Some recent attempts have been made to break down these constructs into their derivative elements, and this will certainly help us to understand where threat management processes end and symptoms begin (Berle and Starcevic, 2005; Coles and Schofield, 2008; Birrell et al., 2011). In particular, viewing clinical anxiety as developing out of an adaptive, evolved system should motivate researchers to distinguish between those elements of their clinical constructs that are adaptive and those that conversely generate symptoms and distress that are typical of clinical disorders.

Three issues related to the nature of the samples used in the experiments reported within this paper are worth commenting upon. Firstly, given analog samples were used in all three experiments, it is not clear how generalizable the conclusions of this paper are to clinical populations. Measures of pre-existing clinical symptoms, IU, inflated responsibility and negative mood were not taken. However, the series of experiments reported in this paper were intended to create a model of the interactions between negative mood, inflated responsibility, and IU within analog samples for potential later extrapolation to clinical samples (Vervliet and Raes, 2013). Further, taxometric studies have suggested that both GAD (Haslam, 2003) and OCD symptoms (Haslam et al., 2005; Olatunji et al., 2008) are generally best considered as dimensional rather than categorical, supporting the appropriateness of studying GAD and OCD related phenomena in analog samples. A second issue with the samples used in the experiments reported is that, whilst males were eligible to participate in all three experiments, the samples obtained for each experiment consisted primarily of females. This raises questions about the generalizability of the results presented to male populations and future research may wish to examine if a similar pattern of results would emerge using predominantly, or exclusively, male samples. A final issue with the samples used in the experiments reported is that the samples consisted almost exclusively of psychology students. This raises questions about the generalizability of the results presented to a wider sociodemographic population and the validity of the results would be strengthened if they were replicated within a wider sociodemographic sample.

There are several further limitations with the studies reported within this paper which should be noted. Firstly, across all three experiments, measures of inflated responsibility, IU and negative mood were taken only after the relevant manipulation and not pre-manipulation to ensure that participants were not alerted to the significance of these factors and the purpose of the experiment prior to the manipulation. The studies therefore lacked the capacity to show an increase or decrease in scores on the relevant measures post-manipulation compared to baseline levels. In all the experiments reported, however, a random sampling assignment process was used that permits the assumption of equality across experimental groups and allows post-manipulation inference of effects of the experimental manipulation on subsequent post-manipulation measures (Campbell, 1957). The measurement approach adopted in this paper has also been used in similar studies in which inflated responsibility (Ladouceur et al., 1995; Bouchard et al., 1999; Arntz et al., 2007) and mood (Scott and Cervone, 2002) have been manipulated. A second limitation with the experiments reported is that measures of psychological constructs which potentially may have affected participants’ responses to the relevant experimental manipulations were not included (e.g., measures of empathy, which may have affected participants’ responses to the vignettes used in Experiments 2 and 3). However, one would hope that the randomization process used in each experiment would result in an equal distribution of participants scoring relatively high or low in terms of these psychological constructs (e.g., empathy) between the relevant experimental groups (Campbell, 1957). Within the series of experiments reported participants taking part in Experiment 1 were not excluded from taking part in Experiment 2, and participants taking part in Experiments 1 and 2 were not excluded from taking part in Experiment 3. It is therefore possible some of the participants taking part in Experiments 2 and 3 may have taken part in a previous experiment and have been somewhat familiar with the experimental procedure and the measures used. Finally, the number of clinical constructs manipulated and measured in the studies reported in this paper was necessarily limited in terms of number. An integrated threat management system as described in this paper would predict that other clinical constructs not manipulated or measured in any of the experiments reported in this paper should also have integrated relationships with other clinical constructs and negative affect as described. Future research may wish to address this possibility.

## Conflict of Interest Statement

The authors declare that the research was conducted in the absence of any commercial or financial relationships that could be construed as a potential conflict of interest.

## Footnotes

1 A growing body of research confirms that many anxiety disorders (e.g., generalized anxiety disorder and obsessive–compulsive disorder) have dimensional rather than categorical properties, indicating a quantitative continuum of processes across non-clinical populations into clinical populations (e.g., Haslam, 2003; Haslam et al., 2005; Olatunji et al., 2008).

2 We have chosen to manipulate and measure negative mood generally in these studies because (1) specific negative moods such as anxiety are difficult to induce in a discrete fashion (Meeten and Davey, 2012), and (2) the constructs selected for investigation in this study are implicated across both anxiety and depression (e.g., Carleton et al., 2012a).
